# Vertical Jump Is Strongly Associated to Running-Based Anaerobic Sprint Test in Teenage Futsal Male Athletes

**DOI:** 10.3390/sports6040129

**Published:** 2018-10-25

**Authors:** Marcelo Magalhães Sales, Ana Paula Maciel, Samuel da Silva Aguiar, Ricardo Yukio Asano, Daisy Motta-Santos, José Fernando Vila Nova de Moraes, Polissandro Mortoza Alves, Patrick Anderson Santos, Lucas Pinheiro Barbosa, Carlos Ernesto, Caio Victor Sousa

**Affiliations:** 1 Departmento de Educação Física, Universidade Estadual de Goiás, Quirinópolis 75860-000, Brazil; apmaciel13@gmail.com (A.P.M.); pmortoza@yahoo.com.br (P.M.A.); 2 Programa de Pós-Graduação em Educação Física, Universidade Católica de Brasília, Brasília 71966-700, Brazil; ssaguiar0@gmail.com (S.d.S.A.); patricksantospas@gmail.com (P.A.S.); Lduarte.barbosa@gmail.com (L.P.B.); ernestobsb@p.gmail.com (C.E.); 3 Departmento de Educação Física, Fundação Municipal de Educação Superior de Bragança, Paulista 12929-600, Brazil; ricardoasano1@gmail.com; 4 Departamento de Esportes, Universidade Federal de Minas Gerais, Belo Horizonte 31270-901, Brazil; daisy@ufmg.br; 5 Colegiado de Educação Física, Universidade Federal do Vale do São Francisco, Petrolina 56304-917, Brazil; josefernando.moraes@univasf.edu.br

**Keywords:** anaerobic metabolism, sprint interval training, soccer

## Abstract

As one of the most popular sport modalities in Brazil, and with an exponential growth in Europe, futsal is characterized by intermittent stimulus of anaerobic high intensity sprints. The running-based anaerobic sprint test (RAST) is one of the most common tests to assess anaerobic power in futsal athletes, however, it presents both time and physical challenges. Therefore, we aimed to correlate RAST with a simpler test, the vertical jump (VJ), in teenage male futsal athletes; Methods: Thirteen volunteers were enrolled and underwent two visits to the laboratory, one for the VJ and the other for the RAST in a randomized order; Results: The association test indicates a strong and significant correlation between VJ and RAST. We conclude that VJ can be used as an alternative to RAST in teenage male futsal athletes.

## 1. Introduction

Futsal is one of the fastest growing sports in the world, being played in more than 100 countries, with millions of players around the world [1]. Data suggest that this sport is the most practiced in Brazil [2].

As far as physical requirements are concerned, futsal is characterized by accelerations and short sprints, usually lasting from one to four seconds and performed at maximum or submaximal intensity [3]. These actions are interspersed by brief periods of recovery, with low-intensity actions or pauses. Approximately 75% of the game actions lasts from one to 18 s, suggesting that anaerobic power may be important for success in this sport [4].

Given these characteristics, the application of performance tests that mimic these actions seems to be important. Among the anaerobic performance tests commonly applied in futsal (involving the ATP-phosphocreatine (PC) and glycolytic metabolisms), we highlight the running-based anaerobic sprint test (RAST), which consists of six 35-m sprints at maximum speed, with 10-s rest intervals between them [5], similar to the athlete’s actions during a futsal match.

On the other hand, despite being a test of easy application, which was the author’s proposal (the title of the paper is: “*Here’s a new running test of anaerobic performance for which you need only a stopwatch and a calculator*”), this test depends on large spaces for its application [6]. In addition, although it is a test of low-cost and easy application, for the evaluation of large groups, such as futsal teams, the test can consume a great amount of time. Not to mention that RAST may expose athletes to greater risks of injury, given the elevated physical demands.

Thus, several studies [7,8,9,10] have sought alternatives to RAST, such as the vertical jump (VJ) [11]. Elegant investigations have shown that there are important associations between repeated sprint tests (RAST and the 30 m sprint test) and the VJ test in several populations [7,8,9,10], and the VJ test is easier to be applied in comparison to RAST. However, to the best of our best knowledge, there are no studies that have investigated the association between the VJ test and RAST in teenage male futsal athletes. 

Based on the above, the aim of the present study was to investigate the level of association between the VJ test and RAST in teenage male futsal athletes. We hypothesized that, similarly to other populations, the VJ test would present an important and significant association with RAST. Thus, exercise physiologists, coaches of futsal teams, and sports scientists, whose research focus on anaerobic performance assessment, and people interested in futsal, would benefit from this research, given the known difficulties of performing RAST.

## 2. Materials and Methods

### 2.1. Sample

After approval from the research ethics committee of the University Center of Gurupi (UNIRG) (approval n. 0183/2010) and their parents or guardians had signed an informed permission form and the subjects given their assent, 13 male amateur futsal athletes, aged 15 to 17 years, participated in the study. Criteria for participation included: (A) Being aged between 15 and 17 years of age; (B) not having any kind of bone, muscle, or joint injury that would preclude performing the experimental sessions; (C) being a member as an athlete in the state local Federation of Futsal in Brazil; and (D) having experience in state-level competitions.

The sample size was calculated to provide a minimum association level of *r* = 0.70, stratified as strong, considering an *α* of 5% (*α* = 0.05) and a *β* of 20% (*β* = 0.20) [12]. Characteristics of the sample are displayed in Table 1 (age, height, body mass, body mass index, VJ peak power, and RAST peak power). 

### 2.2. General Procedures

The experiment consisted of two test sessions (i.e., VJ and RAST) performed in a randomized order at the same time of day with an interval of 48 h between sessions. All procedures were performed as suggested by Draper and Whyte [6]. Anthropometric measurements were carried out before the first test session. All individuals were instructed to come dressed for physical exercise. Therefore, they all came in properly with sneakers and performed the vertical jumping test with their feet shod. It is worth highlighting that all tests were conducted in a sports hall. Furthermore, all subjects were instructed to maintain their eating habits and not perform exhaustive physical activities within 48 h prior to the test sessions.

### 2.3. Anthropometry

Height was measured (SECA^®^ Gmbh, Hamburg, Germany) with the volunteer in a standing position and barefoot, with the ankles, calves, buttocks, scapula, and head leaning on a wall. The position of the head accompanied Frankfurt’s plan, and stature was measured at the moment of inhaling air. Body mass was measured (Toledo 2096 PP, São Bernardo do Campo, Brazil) while the participants wore light clothes. Body mass index (BMI) was calculated as weight (kg)/height (m)^2^.

### 2.4. Running-Based Anaerobic Sprint Test (RAST)

The RAST test consisted of six 35 m maximal runs separated by a period of 10 s of a rest interval. The recorded time was conducted after every effort by a stopwatch (Casio HS-80TW, Shibuya, Tokyo, Japan). Power, in Watts (W), for each sprint was calculated through the product of body mass (BM), in kilograms (kg), and the distance (35 m) raised to the second power. Afterward, this result was divided by the time of each sprint (T), in seconds (s), raised to the third power. As follows: Peak power = body mass (kg) × distance (m)²/time (s)³. The highest power value produced in the sprints was considered the peak power. 

### 2.5. Vertical Jump Test

In this test [11], the subject was instructed to assume the standing position, with the arm extended above the head as high as possible, keeping the soles of the feet in contact with the ground and without flexing the elbows, next to a graduated surface. The participant was asked to mark, with his fingers, the highest position he could reach. To facilitate reading, the fingers of the participants were marked with chalk powder. The test consisted of jumping as high as possible, performing the total extension of the knees, with the balance of the upper limbs for the execution of the jump. At the time of the jump, volunteers could freely flex the lower limbs as well as move the upper limbs to provide the greatest possible vertical thrust. Each individual performed three jumps and the highest jump was considered for peak power calculation [13]. The Sayers power equation [peak power (W) = 60.7 × jump height (cm) + 45.3 × body mass (kg) − 2055] was used to estimate the total power output in Watts [14]. The reliability of the measurements was very high (intra-class correlation coefficient [ICC] = 0.96–0.97). 

### 2.6. Statistical Treatment

The normality of the data was tested by the Shapiro-Wilk test. Variables that did not present normal distribution (age and height) were expressed in medians and their respective interquartile ranges [first quartile (25%) and third quartile (75%)]. Data that presented normality (body mass, body mass index, maximum power in VJ, maximum power in RAST) were expressed as mean and standard deviation. The level of association between the VJ peak power and RAST peak power was analyzed using the Pearson’s correlation coefficient. The sample size was calculated to provide a minimum association level of *r* = 0.70, stratified as strong, considering an *α* = 0.05 and a *β* = 0.20. The reliability of the measurements of the three VJ was carried out using the intraclass correlation coefficient. The level of significance was set at 5% (*p* < 0.05). All procedures were performed using the Software Statistical Package for the Social Sciences for Windows 21.0 (IBM SPSS, Armonk, NY, USA).

## 3. Results

**Figure 1:**
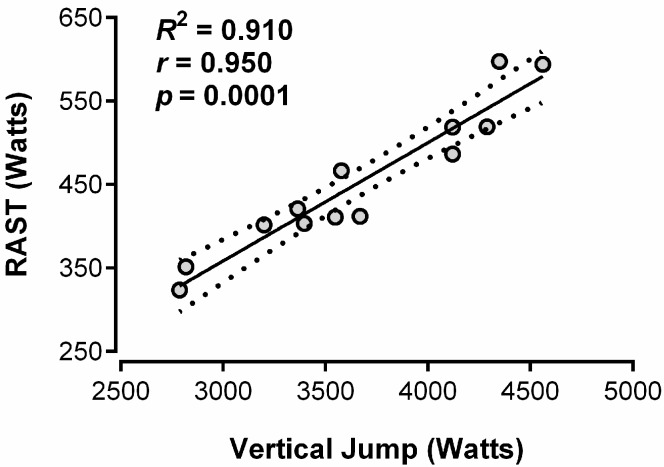
Relationship between the vertical jump (VJ) test and the running-based anaerobic sprint test (RAST); straight line—linear regression; doted lines—95% confidence intervals.

Figure 1 presents the correlation between the VJ test and RAST. The result showed a high and significant correlation (*r* = 0.95; *p* = 0.0001) between the variables. A bivariate equation (including VJ and body mass) was generated to estimate the RAST peak power (R^2^ = 0.91; *p* = 0.0004).

RAST (Watts) = [Vertical Jump (Watts) × 0.133] + [Body mass (kg) × 1.032] − 98.199(1)

## 4. Discussion

The current literature presents few papers that seek to associate speed, acceleration, and maximum power to VJ performance, even though these variables are often observed in sports, such as futsal. Therefore, the objective of the present study was to verify the degree of association of the vertical jump test result and RAST in male teenage futsal athletes. The main finding of the present study (Figure 1) indicates that the VJ test may be an alternative to RAST, given the strong and significant correlation presented between the tests (*r* = 0.95; *p* = 0.0001). 

Our findings are similar to the ones published by Balsalobre-Fernández et al. (2014) who found a strong and significant correlation between the vertical jump test and RAST in professional male basketball players (*r* = 0.73; *p* < 0.001) [9]. Likewise, Davis et al. (2012), showed an even stronger correlation between the countermove jump test and the 40-yard sprint test (*r* = 0.85; *p* = 0.01) in Collegiate Ultimate Athletes [8]. It is worth noting that, although the correlations of the aforementioned studies have different correlation values from the present study (*r* = 0.95; *p* = 0.0001), they are all in the same stratum, representing strong correlations [15].

From a physiological point of view, it is reasonable to hypothesize that the vertical jump test has a strong association with RAST, since the main metabolic pathway required during the actions of both tests are the same (ATP-CP), in addition to the stretching-shortening cycle, which adds the muscular force with the elastic force accumulated by performing a short stretch of the recruited muscles [16,17]. Additionally, intermittent high-intensity sprints can be improved with training that requires an elevated rate of force development, such as jumping training [3]. Thus, suggesting an important association between performance in counter-movement jumps and repeated sprint tests. 

Given the findings of the present study, it is suggested that researchers, coaches, physical trainers, and physiologists of futsal teams, when evaluating anaerobic performance, use the VJ test as an alternative to RAST, since it is a test of easy application and because it requires less time to be performed, allowing coaches to evaluate larger groups in a short time interval. Additionally, it does not need large investments in equipment and large spaces. Finally, the VJ test causes less exposure to the risk of injury because it is a test of lower physical requirement, compared to other test models, such as repeated sprint tests.

The present research has some limitations. Firstly, the original and manual stopwatch model to monitor RAST represents a limitation given the non-automatic method to measure each sprint, although the manual stopwatch has a great reliability when compared to an automated method [18]. Regarding the VJ, one may point out that not using a force platform is a limitation, however, Salles, et al. [19] compared vertical jump tests on the platform with tests performed with the methodology used in the present study, on different days, in 45 soccer players and reported a great reliability between the two methods (ICC = 0.99). We further emphasize that the generated equation was not submitted to validation in the present investigation. The cross-sectional research model of the present study can be characterized as a limitation. Cross-sectional studies have as a main characteristic the achievement of measures in a single moment. Therefore, there is no follow-up period of the individuals, which does not allow us to know what would happen to the variables investigated after a training period. Thus, it is suggested that future studies investigate the effect of strength and power training on the association between the VJ tests and RAST.

## 5. Conclusions

We conclude that VJ can be used as an alternative to RAST in male teenage futsal athletes, if the intention is to analyze the peak power. On the other hand, the results should be interpreted with some caution given the methodologies used to obtain the RAST and VJ results, even though they were considered accurate [18,19]. As a practical application, the results of the present study allow researchers and coaches to evaluate anaerobic performance of similar groups of athletes in less time, making it possible to analyze large groups, such as futsal teams, in shorter periods. Moreover, the VJ test does not require large investments in equipment and does not need large spaces to conduct the tests. It is noteworthy that the VJ test also presents a lower physical requirement than repeated sprinting tests, which may expose this group of athletes to fewer injuries.

## Figures and Tables

**Table 1:** Sample characteristics (*n* = 13). Data expressed as median and interquartile ranges [first quartile (25%) and third quartile (75%)] and mean and standard deviation (±).

Variables	Measures of Position and Dispersion	Normality (*p*)
Age (years)	16.0 (15.0–16.0)	0.0100
Height (cm)	167.0 (163.0–174.0)	0.0365
Body mass (kg)	62.9 ± 6.9	0.2082
BMI (kg∙m^−2^)	21.9 ± 2.0	0.4575
VJ (Watts)	3676.3 ± 574.5	0.5348
RAST (W)	454.4 ± 85.3	0.4684

BMI—body mass index; VJ—vertical jump; RAST—running-based anaerobic sprint test
